# Metabolomic Profiling of the Desiccation-Tolerant Medicinal Shrub *Myrothamnus flabellifolia* Indicates Phenolic Variability Across Its Natural Habitat: Implications for Tea and Cosmetics Production

**DOI:** 10.3390/molecules24071240

**Published:** 2019-03-29

**Authors:** Joanne Bentley, John P. Moore, Jill M. Farrant

**Affiliations:** 1 Department of Molecular and Cell Biology, University of Cape Town, Private Bag, Cape Town 7701, South Africa; jill.farrant@uct.ac.za; 2 Institute for Wine Biotechnology, Department of Viticulture and Oenology, Faculty of AgriSciences, Stellenbosch University, Matieland 7602, South Africa; moorejp@sun.ac.za

**Keywords:** *Myrothamnus flabellifolia*, desiccation tolerance, LC-MS/MS, flavonoids, anthocyanins, resurrection plant, phenolics

## Abstract

The leaves and twigs of the desiccation-tolerant medicinal shrub *Myrothamnus flabellifolia* are harvested for use in traditional and commercial teas and cosmetics due to their phenolic properties. The antioxidant and pharmacological value of this plant has been widely confirmed; however, previous studies typically based their findings on material collected from a single region. The existence of phenolic variability between plants from different geographical regions experiencing different rainfall regimes has thus not been sufficiently evaluated. Furthermore, the anthocyanins present in this plant have not been assessed. The present study thus used an untargeted liquid chromatography-tandem-mass spectrometry approach to profile phenolics in *M. flabellifolia* material collected from three climatically distinct (high, moderate, and low rainfall) regions representing the western, southern, and eastern extent of the species range in southern Africa. Forty-one putative phenolic compounds, primarily flavonoids, were detected, nine of which are anthocyanins. Several of these compounds are previously unknown from *M. flabellifolia.* Using multivariate statistics, samples from different regions could be distinguished by their phenolic profiles, supporting the existence of regional phenolic variability. This study indicates that significant phenolic variability exists across the range of *M. flabellifolia*, which should inform both commercial and traditional cultivation and harvesting strategies.

## 1. Introduction

Plants have been the subjects of phytochemical investigations due their production of a diversity of phytonutrients, many of which are valued for their antioxidative properties. Numerous studies have focused on beneficial plant-derived beverages; two common examples include red wine and tea. It has been established that the phenolics present in plant extracts as secondary metabolites, including phenols, phenolic acids such as hydroxycinnamic and hydroxybenzoic acid derivatives, and flavonoids, contribute inordinately to the overall antioxidant capacity and towards protection against pathogens and UV radiation [1]. The dietary intake of these phenolic compounds has been associated with lower rates of cancer [2], cardiovascular disease [3], and diabetes [4]. 

**Figure 1:**
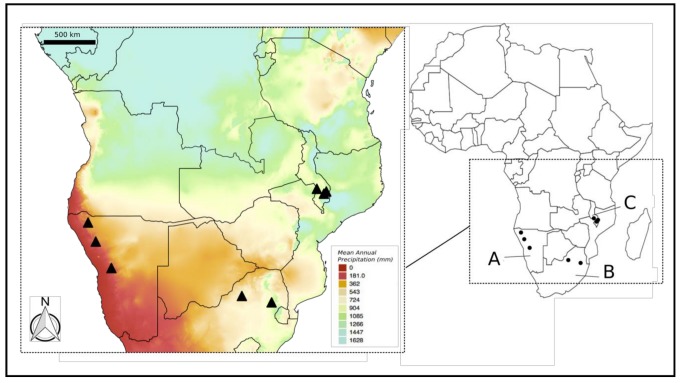
Map of mean annual precipitation (MAP) based on BIOCLIM variable 12 from www.worldclim.org/bioclim indicating the countries referred to in the text; **A**: Namibia, **B**: South Africa, and **C**: Malawi. The sampling sites in the present study are also indicated by black triangles. The map was produced in Quantum (Q) GIS v. 2.14 “Essen” (https://qgis.org/en/site/).

*Myrothamnus flabellifolia* Welw. (Gunnerales, Myrothamnaceae) is a medicinal shrub that is distributed in the summer rainfall regions of southern Africa. Across its distribution range, it occurs in both high and very low rainfall regions (Figure 1). The plants grow on isolated “inselbergs” or rocky outcrops where they have colonised a unique, but harsh, niche [5]. In order to thrive in this niche, which experiences high water runoff and increased rates of evaporation, *M. flabellifolia* has evolved the trait of desiccation tolerance. This allows it to tolerate dehydration to an air-dry state, enduring up to 95% cellular water loss for an extended time period following which metabolism is rapidly recovered in the tissues within 24–72 h of rehydration [6,7,8]. Plants exhibiting this vegetative desiccation tolerance are collectively known as “resurrection plants”. As a homoiochlorophyllous plant, *M. flabellifolia* maintains its photosynthetic apparatus during desiccation but responds to the desiccation-induced reactive oxygen species (ROS) damage by mobilising free-radical scavenging systems and producing anti-oxidative and protective metabolites [9]. However, chloroplasts themselves constitute cellular sources of ROS, and the production of phenolics, including anthocyanins, restricts excess light absorption when the plants are in the dry state by masking the chlorophyll, therefore acting as antioxidants [10]. Anthocyanins, as flavonoids, are also believed to decrease the osmotic potential of the leaves and reduce stomatal conductance, thereby minimising water loss via transpiration under hot conditions [11,12]. Anthocyanins are also valued for their wide range of biological activities, including antioxidative and anti-inflammatory [13], anticancer [14], and others [15].

In addition to its traditional use as a medicinal preparation for the treatment of chest infections, uterine pain, and gingivitis, *M. flabellifolia* is also used in commercial tea products (typically marketed as “resurrection tea”) as well as cosmetics (e.g., Giorgio Armani’s Crema Nera range). Its phytochemical value has been verified in numerous studies; for instance, 3,4,5-tri-*O*-galloylquinic acid, isolated from *M. flabellifolia*, was shown to protect membranes from desiccation-induced ultrastructural damage from ROS [16] and also inhibited viral reverse transcriptases [17]. A galloyl glucose hexahydroxydiphenic acid isolated from the same plant was found to inhibit the growth of triple negative breast cancer cells [18], while a proanthocyanidin-rich extract was demonstrated to be active against herpes simplex virus 1 [19]. Additionally, an extract from *M. flabellifolia* was also found to inhibit α-glucosidase and α-amylase activities, thereby indicating promising anti-diabetic activity [20]. 

The total phenolic content of South African and Namibian *M. flabellifolia* material was previously found to constitute more than 50% and 70% of the dry weight, respectively, represented largely by the membrane-protectant 3,4,5-tri-*O*-galloylquinic acid [21]. The authors attributed the variability in the content of this compound to habitat differences, suggesting that the drier and hotter environment of the Namibian plants would select for greater quantities of this protective compound. Numerous other phenolic compounds have also been identified in *M. flabellifolia*, including an arbutin derivative (2,3-di-*O*-galloylarbutin) [22]; quercetin, as well as its 3-*O*-β-d-galactosides, -glucosides, -glucuronides, and 3-*O*-α-l-rhamnosides; mono-, di-, and tri-galloylated flavonol glycosides; and various galloylquinic acid derivatives and ellagitannins [23]. Various flavan-3-ols (epicatechin, epigallocatechin, and their 3-*O*-galloylated analogues), procyanidins, and proanthocyanidins [24,25] were also isolated and characterised. However, the material used in these investigations originated from a single region, namely South Africa (Figure 1). One study [26] assessed the phytochemical constituents of Namibian *M. flabellifolia* material and indicated the presence of flavonoids, anthocyanins, alkaloids, steroids, terpenoids, triterpenes, cardiac glycosides, saponins, phlobatannins, tannins, and polyphenols in the leaves, but these were neither identified nor quantified. Koonjul et al. [27] extracted anthocyanins from the leaves of *M. flabellifolia* from South Africa and detected cyanidin and delphinidin spectrophotometrically, but these were not further classified. 

A global assessment of the phenolic constituents, including anthocyanins, present in *M. flabellifolia* from across its geographic range is currently lacking. As the production of secondary metabolites, and phenolics in particular, is typically influenced by environmental conditions, it can be expected that plants from different regions would exhibit different phenolic profiles. Should phenolic variability exist across its range, it would have implications for the various commercial tea and cosmetic products from this species. An evaluation of the variability is therefore warranted given the widespread commercial and traditional use and increasing popularity of products from this plant. We thus used an untargeted liquid chromatography-quantitative time-of-flight-tandem-mass spectrometry (LC-QTOF/MS) metabolomics approach to assess any regional differences in phenolic compounds, including anthocyanins, from plants sampled directly in the field and hypothesised that the plants from different regions would be associated with distinct phenolic profiles. Eight populations representing the western (low rainfall), southern (moderate rainfall), and eastern (high rainfall) range of the species distribution were included (Figure 1). Putative phenolic compounds were identified based on their MS^E^ spectra in both the negative ionisation and positive ionisation (for anthocyanins) modes. Using this information, multivariate statistics were used to compare the phenolic profiles of the different populations in order to ascertain if plants from the different regions were associated with any particular phenolic signature. In addition, total phenolics and total anthocyanins were also assayed and compared between the populations. The findings of this study should inform future cultivation and harvesting strategies. 

## 2. Results

### 2.1. Total Anthocyanins and Phenolics

**Figure 2:**
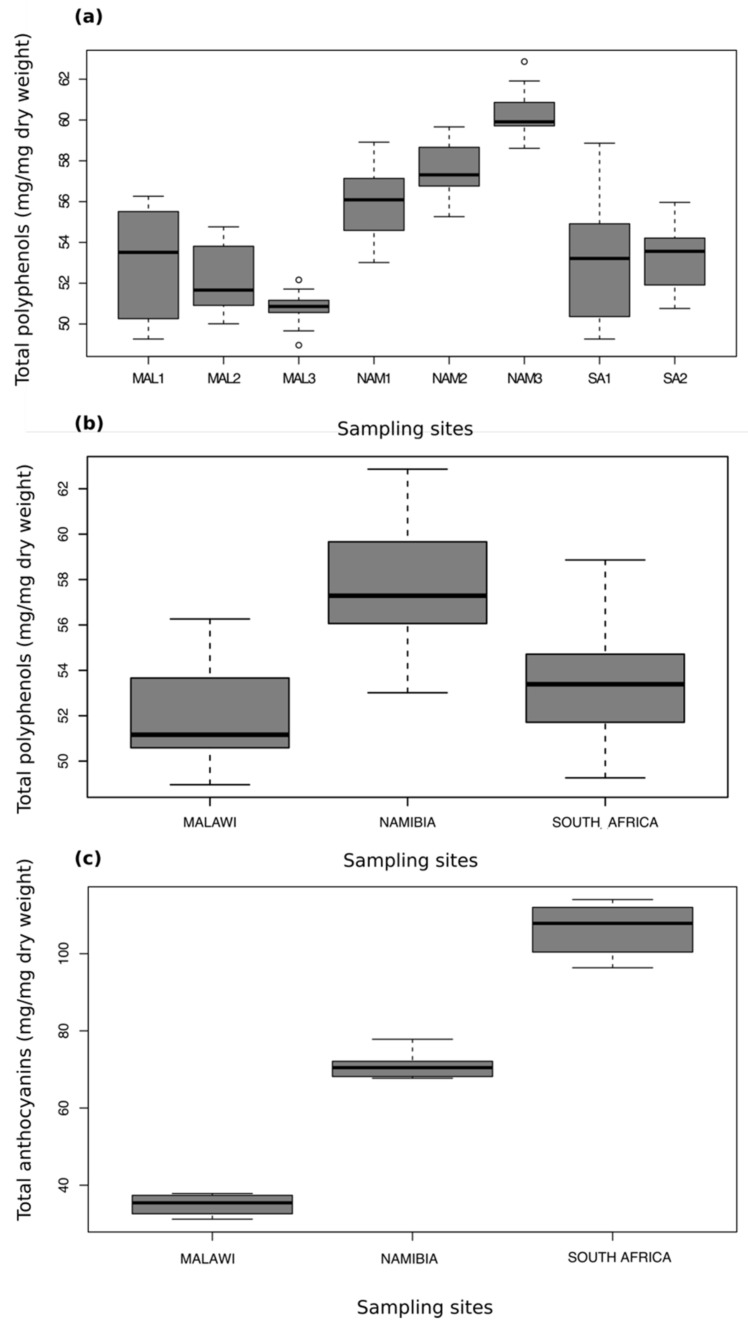
(**a**) Box plot showing the results of the total polyphenolics assay between samples collected from all sampling sites. Analysis of variance (ANOVA) followed by Tukey’s post-hoc tests were used to assess significant interactions at *p* < 0.05. No significant differences in polyphenols were detected between samples within the same country (Malawi, South Africa, and Namibia). The Namibian samples possessed significantly higher polyphenolics than all the Malawian samples and all of the South African samples, with the exception of the interaction between NAM1 (“Etusis”) and SA2 (“Waterberg”). (**b**) Box plot showing the results of the total polyphenolics assay with the samples grouped by country. The Namibian samples possessed significantly higher phenolics than both South Africa and Malawi, whereas South Africa and Malawi did not differ significantly from one another. (**c**) Box plot showing the results of the total anthocyanins assay between samples pooled by major area (country). All interactions were determined to be significant at *p* < 0.05.

Total phenolics and anthocyanins are shown as box plots in Figure 2. The Namibian samples were determined to have a significantly higher phenolic content (F = 16.25, df = 7, *p* < 0.001) than both the South African and Malawian samples, with the exception of the relationship between “Waterberg” (South Africa) and “Etusis” (Namibia) (*p* > 0.05). A different pattern was observed with regards to total anthocyanins, whereby the South African samples had the highest anthocyanin contents, followed by the Namibian samples and then the Malawian samples (F = 322.2, df = 2, *p* < 0.001).

### 2.2. Tentative Identification of Anthocyanins in the Positive Ionisation Mode 

**Figure 3:**
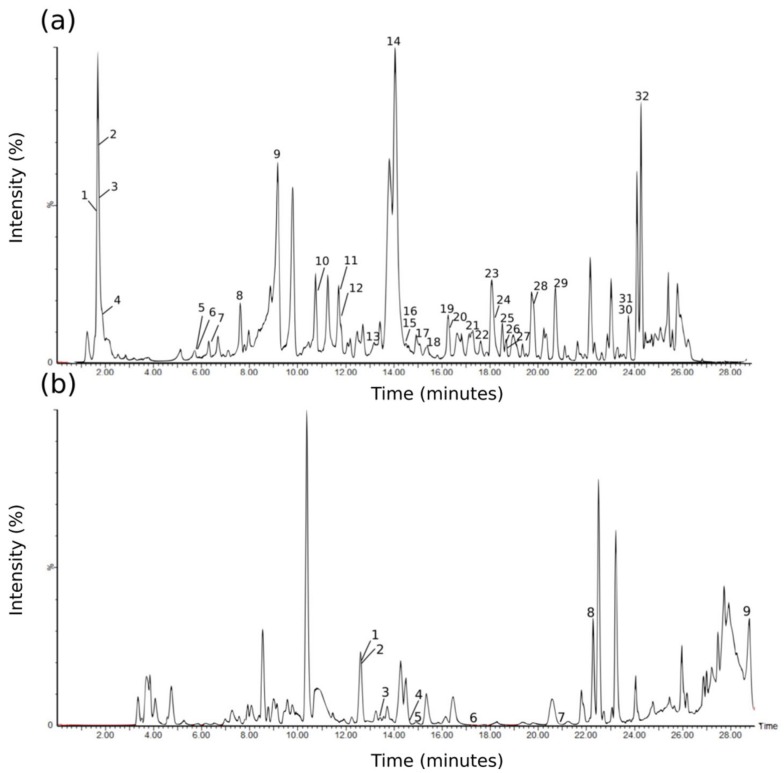
(**a**) Base peak intensity (BPI) chromatogram of the negative ionisation analysis. (**b**) BPI chromatogram of the positive ionisation analysis. The chromatograms are a representative sample from a locality in Namibia (“Etusis”).

While the presence of anthocyanins, including delphinidin and cyanidin, was previously detected in *M. flabellifolia* by means of spectrophotometric measurement at 547 and 558 nm [27], these have not been elucidated using an MS/MS approach. Eight anthocyanins were tentatively identified in this study, thus constituting a new finding. The anthocyanins detected in the positive ionisation mode are listed in Table 1, where their *m*/*z* values, MS/MS values, and retention times are listed, and the corresponding chromatogram indicating the various peaks is provided in Figure 3. Several derivatives of delphinidin and cyanidin, as well as petunidin and peonidin, were putatively identified in this study (Table 1). Delphinidin-3-*O*-glucoside (*m*/*z* 465) was tentatively detected in the positive ionisation spectrum (compound **1**) and exhibited the characteristic fragmentation pattern [303 (aglycone), 257, 229]. The ion at *m*/*z* 611, represented by compound **2**, could possibly be one of three molecules based on an assessment of the fragmentation spectra (465, 303, 153). It might represent either delphinidin-3-(6-*O*-coumaroyl)glucoside, or delphinidin rutinoside, or quercetin 3-*O*-rutinoside (rutin). Further investigation of the fragmentation spectra, or comparison with authentic standards, is needed to confirm the identification. Compound **4** was deduced to represent cyanidin-3-*O*-rutinoside at *m*/*z* 595 with fragment ions at 449 and 287 (aglycone). Compound **5** was believed to represent delphinidin-3-*O*-arabinoside, with an *m*/*z* of 435 and the fragment aglycone ion at 303. Cyanidin-3-*O*-arabinoside was ascribed to compound **6** (*m*/*z* 419) and a characteristic aglycone fragment at 287. Compound **7** might represent peonidin-3-*O*-glucoside/galactoside (*m*/*z* 463) with a dominant 301 aglycone fragment present in the spectrum. Petunidin-3-*O*-arabinoside was tentatively identified to represent compound **8**, with a dominant characteristic fragment ion at 317. Delphinidin-3-acetylglucoside was assigned to compound **9** (*m*/*z* 507), and characteristic fragments at 463 and 303 (aglycone) were observed. 

### 2.3. Tentative Identification of Phenolic Acids and Derivatives, Including Hydrolysable Tannins, in the Negative Ionisation Mode

The phenolic compounds detected in the negative ionisation mode are listed in Table 1, where their *m*/*z* values, MS/MS values, and retention times are listed, and the corresponding chromatogram indicating the various peaks is provided in Figure 3. Quinic acid (compound **1**), a cyclohexanecarboxylic acid, was dominant in all the spectra and was identified based on the 173 (quinic acid–H–H_2_O) and 133 fragment ions of the *m*/*z* 191 peak. Compounds **3** and **4** were thought to represent quinic acid derivatives due to the presence of the *m*/*z* 191 ion. Compound **5** is an unknown phenolic with an *m*/*z* of 317 and fragments of 191 and 135. This peak eluted at the same time as another, more dominant, peak (possibly arbutin), which made the spectrum challenging to interpret. Further chromatographic separation of these two peaks is required. The glycosylated hydroquinone arbutin (4-hydroxyphenyl-β-glucopyranoside) (compound **6**) was represented by *m*/*z* 271 and fragments at 161, representing the loss of the hydroquinone, and 109. Compound **7** was ascribed to gallic acid (also known as 3,4,5-trihydroxybenzoic acid) with an *m*/*z* of 169 and typical fragment ion of 125 (dihydroxy phenol moiety). Compound **8** was putatively identified as 3/4/5-*O*-galloylquinic acid (*m*/*z* 343) based on the 191 (quinic acid), 173 (quinic acid–H–H_2_O), 169 (gallic acid), and 125 (dihydroxy phenol moiety) fragments. Compound **9** was believed to represent a quinic acid derivative with an *m*/*z* of 405 and fragments of 191 (quinic acid) and 173 (loss of a water molecule). Compound **10**, with an *m*/*z* of 495 and fragments at 343, 325, 191 (quinic acid), and 169 (gallic acid), was assigned to 3,4/3,5-di-*O*-galloylquinic acid. Methyl gallate (a methyl ester of gallic acid) was thought to represent the compound at *m*/*z* 183 (compound **11**), with a characteristic fragment at 124. Compound **12** was estimated to represent another quinic acid derivative with an *m*/*z* of 423 and predominant fragments of 257 and 191. The next identified compound at **13** probably represented trigalloylglucose with an *m*/*z* of 635 and a dominant fragment at 465; representing the loss of an amu of gallic acid. 3,4,5-Tri-*O*-galloylquinic acid (compound **14**) was identified from its typical fragment ions at *m*/*z* 495, 343, 191, and 169, a pattern that is characteristic of a trigalloyl substitution of a quinic acid core. Compound **15** represents an unknown phenolic at *m*/*z* 431. Compound **16** was thought to represent gallocatechin/epigallocatechin gallate (*m*/*z* 457) based on the 305 (epigallocatechin moiety), 169, and 125 fragments, and was previously documented in *M. flabellifolia* [24]. Compound **19** probably represents a penta-galloylquinate ester at *m*/*z* 937 with fragments at 799, 468, 301, and 169. This peak indicates a higher molecular mass gallic acid polyester that was previously determined to be the result of the multiple galloylation of the central 3,4,5-tri-*O*-galloylquinic acid core [21] and is typically present in much smaller quantities than 3,4,5-tri-*O*-galloylquinic acid. Ellagic acid (compound **23**), which is the dilactone of hexahydroxydiphenic acid, was tentatively identified with an *m*/*z* of 301, with the MS/MS spectrum of the deprotonated molecule indicating the expected fragmentation with *m*/*z* 229 and *m*/*z* 169. Ellagic acid has previously been detected in *M. flabellifolia* [28]. Another compound (**22**) could not be identified but appeared to be an ellagic acid derivative based on the presence of the *m*/*z* 301 fragment. The ion at *m*/*z* 483 (compound **28**) was believed to represent digalloylglucose based on the fragmentation spectrum of 301 (loss of glucose and galloyl groups), 271, and 169 (gallic acid). Four additional phenolic compounds that could not be identified are represented by compounds **17**, **18**, **26**, and **29**, with fragment ions at 191 (quinic acid) and 169 (gallic acid). 

### 2.4. Tentative Identification of Flavonoids in the Negative Ionisation Mode

The spectrum associated with compound **2** was difficult to interpret, but this ion was thought to represent either an isomer of arbutin or the flavonoid naringenin based on the *m*/*z* of 271 and the 161, 151, 125, and 119 fragments. Compound **21** was suggested to represent quercetin-3-*O*-hexose-gallate, with an *m*/*z* of 615 and fragment ions at 463, 300, 169, 151, and 125. Compound **24** was attributed to quercetin-glucoside (*m*/*z* 463) with a fragment at 301 (aglycone fragment). Quercetin-3-*O*-glucuronide, or miquelianin, was tentatively identified as **25**, with an *m*/*z* of 477 and characteristic fragment ions at 301 (aglycone fragment), 273, 257, 193, 179, and 151 and was previously found to have potential as a barcode for distinguishing *M. flabellifolia* material collected from Malawi and South Africa from material collected in Namibia [29]. Compound **26** (*m*/*z* 469) could not be identified but was supposed to possibly represent a quercetin derivative due to the presence of a fragment ion at 301 representing the radical anion of the aglycone quercetin. Galloyl quercetin-3-*O*-rhamnoside (compound **27**) was putatively identified based on the characteristic fragment ions at 447, 301 (aglycone quercetin), and 171. Quercitrin [(quercetin 3-*O*-rhamnoside) compound **30**] was identified based on the ion at *m*/*z* 447 and fragments at *m*/*z* 301 (aglycone fragment), 300 (radical aglycone anion), 271, 255, and 179 and constitutes a potential barcode for distinguishing Namibian *M. flabellifolia* material from the material from other regions [29]. Another compound with an *m*/*z* of 447 co-eluted with quercitrin and was deduced to represent methylellagic acid pentose based on the presence of the *m*/*z* 315 ion in the spectrum. Compound **32**, deduced to represent isorhamnetin (3-*O*-methylquercetin), had an *m*/*z* of 315 and characteristic fragment ions of 300 (release of a methoxy group), 271, 255, 243, 227, 199, and 151. The flavonol quercetin-7-*O*-glucuronide (compound **3** in the positive ionisation spectrum) was putatively detected with an *m*/*z* of 479 and a 303 fragment in the positive ionisation spectrum.

### 2.5. Comparison of the Phenolic Profiles

**Figure 4:**
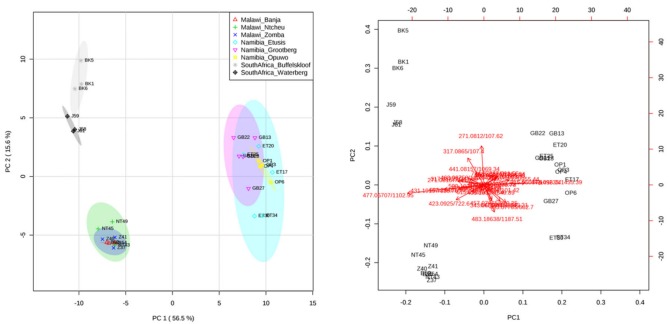
Principal components analysis (PCA) scores plot (left) and loadings plot (right) based on the identified phenolics from the negative ionisation analysis. Fourteen samples from Namibia, nine samples from Malawi, and six samples from South Africa were included in the PCA.

The data dimensionality reduction technique principal components analysis (PCA) was performed on the phenolic compounds from the negative ionisation analysis (Table 1), and the results are indicated in Figure 4. This was done to visualise the population-level differences in the phenolic profiles. The first principal component explained 56.5% of the variability in the dataset, while component two accounted for 15.6%, together accounting for the acceptably large percentage of 72.1 The Namibian samples all clustered together and indicated significant overlap, as did the Malawian samples, while the South African samples were much more variable. The findings of the PCA suggest that plants from different regions are characterized by particular phenolic profiles, which confirms speculations from earlier work [16] and has implications for the standardization of the medicinal material and products from this plant. The phenolic signature from the PCA also corroborates the total phenolics and anthocyanins assays, in that the three populations could also be distinguished based on the assay results. According to the PCA loadings plot, the ion contributing most to the separation of the South African and Malawian samples from the Namibian samples was compound **25** (miquelianin). The ion contributing most to the separation of the Namibian samples from the other samples was compound **30** (quercitrin). These findings corroborate our earlier study [29].

One-way analysis of variance (ANOVA) with Fisher’s Least Significant Difference (LSD) post-hoc tests were used to determine the ions that differed significantly between the different populations. At *p* < 0.05, all compounds, with the exception of compounds **9**, **19**, **27**, and **29**, differed significantly between the different sampling sites. We thus selected a stricter *p*-value cut-off of <0.001 to identify highly significant relationships (Table S1, Figure S1). A total of 15 ions were found to differ significantly between the different regions at *p* < 0.001 and are discussed below in order of significance. Compounds **30**, **25**, and **32**, respectively representing quercitron, miquelianin, and isorhamnetin, differed significantly between the regions and were also identified as potential biomarkers in our previous chemometric study [29]. An unknown phenolic (compound **15**) was highly abundant in the samples from Waterberg (South Africa). A naringenin or arbutin isomer (compound **2**) was significantly more abundant in the samples from South Africa, whereas 3,4,5-tri-*O*-galloylquininc acid (compound **14**) was low in abundance in the samples from South Africa and one location in Namibia (Opuwo). Three unknown phenolics (compounds **3**, **5**, and **12**) were highly variable between the regions. Compound **4**, which might represent a dimer of quinic acid or a caffeic acid glycoside, was very low in abundance in the Namibian samples, as was arbutin (compound **6**). In contrast, ellagic acid (compound **23**) was significantly highly abundant in the Namibian samples, as was compound **10** (3,4/3,5-Di-*O*-galloylquinic acid), which was low in abundance in the South African samples. A similar result was found with 3/4/5-O-galloylquinic acid (compound **8**). Compound **24**, believed to represent quercetin glucoside, varied substantially between the regions.

## 3. Discussion

An assessment of the phenolic compounds present in *M. flabellifolia* indicates that the plant possesses significant potential as a source of phenolic antioxidants. In this study, 41 phenolic compounds, including eight anthocyanins, were tentatively identified from *M. flabellifolia* leaf material originating from eight populations sampled from across three major geographic regions using LC-MS/MS metabolomics (Table 1). Several of these compounds have previously been detected in *M. flabellifolia*, including compounds **1**, **6**, **7**–**9**, **13**–**16**, **19**, **23**, **25**, **28**, **30**, and **32** obtained from the negative ionisation analysis (Figure 3, Table 1). The compounds detected in the positive ionisation mode (nine compounds in total) have also not previously been reported from *M. flabellifolia*. The remaining phenolic compounds, based on an assessment of the literature, have not been previously documented in *M. flabellifolia*. A PCA based on these phenolic compounds (Figure 4) indicated that the samples grouped into regions based solely on their phenolic profiles, particularly the samples from Namibia and Malawi, strongly supporting the hypothesis that plants from different regions are characterized by particular phenolic profiles and implies that a particular suite of phenolics is required for survival in these different environments. This confirms earlier work by Moore et al. [16] and has implications for the standardization of the medicinal material and products from this plant. Furthermore, the total phenolics and anthocyanins assays corroborated the phenolic signature, in that the three populations could also be distinguished based on the assays results. As we were unable to include the putative anthocyanins in our metabolomic comparison of the populations, some caution should be taken when interpreting the results. However, our earlier fingerprinting analysis [29] based on a global metabolomics dataset, and not just the phenolics, was able to discriminate between the different populations, suggesting that the metabolite signatures of the samples from the different regions are relatively robust.

The higher total phenolic content of the Namibian populations (Figure 2) supports the findings of a Moore at al. [16] who also discovered that the Namibian samples had significantly higher total phenolic content than the South African samples, although their analysis did not include samples collected from the eastern region. In contrast, the results of the total anthocyanin assay (Figure 2), in which the South African samples exhibited the highest anthocyanin contents, were unexpected given the significantly greater phenolic concentration detected in the Namibian samples, not only in this study. One explanation may be that anthocyanin synthesis and degradation is metabolically expensive [11], particularly in the context of the comparatively shorter growing season (shorter rainfall periods) of *M. flabellifolia* in Namibia. This, of course, does not explain why anthocyanin contents were lowest in Malawi. An alternative, and more promising, explanation, is related to frost tolerance. The South African populations, particularly from Buffelskloof, occur within a region that experiences frequent winter (dry season) frost, while this occurrence is very rare in the other regions. Anthocyanins have previously been found to be associated with frost tolerance; for instance, Arabidopsis mutants deficient in frost tolerance were unable to accumulate anthocyanins [30]. Additionally, northern ecotypes of *Populus*, which are required to survive colder winters than the southern ecotypes, accumulated more anthocyanins [31]. This hypothesis is interesting and warrants further investigation. While the presence of anthocyanins, including delphinidin and cyanidin, was previously detected in *M. flabellifolia* by means of spectrophotometric measurement at 547 and 558 nm [27], these have not been elucidated using an MS/MS approach. The eight putative anthocyanins identified in this study are thus a new finding. Several other phenolic compounds were also detected in this study that have not been previously described from *M. flabellifolia*. For instance, methyl gallate (compound **11**, Table 1), a methyl ester of gallic containing two pairs of catecholic moieties, has been shown to act as a powerful antioxidant and inhibitor of lipid peroxidation and DNA damage [32]. It is prevalent in the plant kingdom, particularly in medicinal plant extracts, and was most abundant in the Namibian samples, but occurred at lower concentrations in the samples from the other regions. A previous study found an extract of *M. flabellifolia* to strongly inhibit herpes simplex virus type 1 [19], which may be attributed at least in part to the presence of methyl gallate, as this compound has been found to be active against herpes simplex virus type 2 [33].

Several of the identified phenolics are valuable phytochemicals present in other teas and extracts used in cosmetics. Gallocatechin/epigallocatechin gallate (compound **16**), previously documented in *M. flabellifolia* [24], was present at low concentrations in the South African populations. It is one of the primary phenolic constituents of green tea and is valued for its various phytochemical applications, including chemoprotective, anti-neurodegenerative, antioxidant, anti-HIV, and antibacterial properties, to name a few. This would therefore also influence the beneficial properties of tea and other preparations from *M. flabellifolia*. In terms of the ions that were highly significantly different between the regions, ellagic acid (compound **23**), also previously detected in *M. flabellifolia* [28], was most abundant in the Namibian samples. This compound has been shown to act as a potent antioxidant in red wines [34] and thus may have implications for the efficacy of the tea obtained from different *M. flabellifolia* populations. Similarly, quercetin glucoside, which varied significantly between the regions, is also present in different quantities in oolong, black, and green teas [35]. 3,4,5-Tri-*O*-galloylquinic acid, in addition to showing anti-viral reverse transcriptase activity [17], was also found to act as a membrane protectant [21], which has prompted much interest into the use of *M. flabellifolia* extracts in anti-aging cosmetics. Arbutin, also differing significantly between the sample sites, has long been known to act as an inhibitor of melanogenesis, which has fostered interest in its use in cosmetics [36]. The variability in these compounds between the populations would thus influence the proposed phytochemical value of the cosmetics produced from extracts of this plant. The possible discovery of naringenin (compound **2**), which was significantly higher in the South African extracts and was also previously found to have possible utility as a barcode for distinguishing the South African *M. flabellifolia* material from the Malawian material, is interesting, as naringenin is required for the synthesis of various flavonoids, including anthocyanins, which also substantiates the findings of the total anthocyanins assay whereby South African samples possessed the highest total anthocyanins. In summary, our findings demonstrate that *M. flabellifolia* exhibits significant phenolic variability across its range, and even within the same country, which has direct implications for the food and cosmetics industries that use extracts from this plant. Whether this variability is genotypically- or environmentally-controlled, or both, requires further exploration.

## 4. Materials and Methods

### 4.1. Plant Material and Sample Preparation

A total of eight populations from three major geographic regions representing the eastern (Malawi: three populations), western (Namibia: three populations), and southern range (South Africa: two populations) of the species were sampled (Figure 1). Between three and seven representative individuals from each of the eight populations were sampled for the LC-MS/MS analysis (metabolomic sampling information for both negative and positive ionisation modes is provided in Table S2) as follows: Namibia [Etusis (22°10′3.14″S, 15°44′42.64″E) n = 7; Grootberg (19°44′1.23″S, 14°18′25.40″E) n = 7; and Opuwo (17°58′54.89″S, 13°35′59.99″E) n = 4], Malawi [Ntcheu (14°52′22.73″S, 34°39′37.36″E) n = 4; Zomba Plateau (15°19′35.26″ S, 35°18′26.75″ E) n = 3, and Banja Hill (15° 7′24.59″S, 35°31′47.89″E) n = 4, and South Africa [Waterberg (24°44′18.13″S, 27°45′24.48″E) n = 4, and Buffelskloof (25°19′39.13″S, 30°29′41.80″E) n = 3]. A field-based extraction procedure was used to directly quench metabolism in the field [37]. Further details relating to the sampling sites, including the climatic characteristics, and the methods are provided in our earlier study [29]. Briefly, the samples were dried-down upon return to the laboratory and then later resuspended in the appropriate volume of methanol at the same concentration prior to analysis. For the total phenolics and anthocyanins assays, leaf material that had been collected in the field from the aforementioned populations and dried on silica gel was used. For the total phenolics assay, material from each of the eight collection sites was used. Due to a lack of sufficient plant material, the anthocyanins assay was performed on plant leaf material that had been pooled for each country (i.e., Namibia, South Africa, and Malawi). This was reasonable because the preliminary metabolomic results indicated that the samples from each of the countries grouped together.

### 4.2. Total Phenolic and Anthocyanin Assays

Total phenolic contents of triplicate samples of 20 mg leaf material from each subpopulation were estimated using Folin–Ciocalteu reagent [38] based on a previous protocol [39]. For the anthocyanin assay, pooled leaf material from the populations was used following a previous protocol [40]. Six biological replicates were used from each country and the samples were assayed in triplicate.

### 4.3. LC-MS/MS Parameters

In preparation for the metabolomics analysis, the dried samples were made to the same concentration by calculating the soluble solid mass and then resuspending in the appropriate amount of methanol (ROMIL Ltd. Cambridge, UK; HPLC grade). The tubes were centrifuged twice at 14,000 rpm at room temperature for 5 min each time, with the supernatant carefully collected and pooled after each spin in order to obtain a clear solution. A biological pooled quality control (QC) sample consisting of 5 µL of each sample extract was used to validate the repeatability of the metabolomics dataset. In addition, one random technical replicate was performed during the analysis as an additional verification to the QC samples. Negative ionisation mode was used to detect the phenolic compounds. The samples were injected into the MS in a random fashion as determined using the “RAND” function in Microsoft Excel, with the exception that the analysis started with three blank methanol controls and a QC sample, with the QC samples being injected every 15th sample. The LC-MS/MS operating conditions had previously been optimised by the Central Analytical Facility (CAF; Stellenbosch University, Stellenbosch, South Africa) for the targeting of phenolics. For the negative ionisation analysis, the procedure and equipment were identical to that discussed in a previous study [29]. As anthocyanins are generally detected in the positive ionisation mode [41], this mode was thus selected for the detection of anthocyanins. The operating conditions of the positive ionisation mode analysis differed to that of the negative ionisation phenolics analysis. Specifically, 3 μL of each sample was injected at 0.4 mL/min with the following 32-min solvent gradient: 100% solvent A (97% water, 3% acetonitrile and 0.1% formic acid) for 2 min, followed by an 18 min gradient to 40% solvent B (90% acetonitrile, 10% water and 0.1% formic acid), and then a 5 min gradient to 100% solvent B, which was held for 5 min before a 2 min gradient to 100% solvent A. The column was a Chromolith^®^ HighResolution RP-18 end-capped 100-4.6 (Merck, Burlington, MA, USA) coupled with a Waters (Waters, Milford, MA, USA) Acquity ultra-performance (UP)LC and photo diode array detector fitted to a Waters Synapt G2 Q-TOF MS. Due to cost, time, and equipment constraints, only three samples (two from Namibia and one from Malawi) were subjected to LC-MS/MS analysis. Alternatively, LC-MS scans (i.e., not in MS/MS mode) were conducted for more samples (one from South Africa, three from Malawi, and three from Namibia). The compounds detected in these scans were identified by comparison with the MS/MS results. For the LC-MS scans, 5 μL of each sample was used, and the chromatography system was an Agilent (Agilent Technologies, Santa Clara, CA, USA) 1290 Infinity HPLC system coupled to an Agilent 6530 Accurate-Mass Q-TOF fitted with an Agilent JetStream Electrospray Ioniser (ESI) in the Mass Spectrometry Unit of the Molecular and Cell Biology Department, University of Cape Town (Cape Town, South Africa). The same solvent gradient and column as detailed above were used. Accurate mass spectra were collected in the 100 to 1700 *m*/*z* range, with mass calibration reference masses of 121.0509 and 922.0098.

### 4.4. Multivariate Statistical Analysis

ANOVA followed by Tukey’s honest significant difference (HSD) post-hoc tests were used to evaluate whether there were any significant differences in total phenolic and anthocyanin content between the different regions/subpopulations as determined by the assays. The data were first assessed for normality and homogeneity of variance. This was performed in the R statistical environment [42] and the ggplot2 package [43] used to construct graphs. For the MS/MS assessment, the spectra were viewed in MassLynx, and phenolic compounds were putatively identified based on an examination of the fragmentation spectra. When available, MS/MS spectra from the METLIN database (https://metlin.scripps.edu/) and published studies were used for comparison. As the compounds were tentatively identified based on their precursor ions and fragmentation spectra in the absence of analytical standards, the identifications correspond to level 2 (for known compounds) and level 3 (for those compounds identified only as being phenolic in nature) in the Metabolomics Standard Initiative guidelines for minimum reporting standards [44]. For the multivariate statistical comparisons of the putatively identified phenolic compounds between the different regions, the CDF files were processed using the “xcms” package [45,46] in R. Briefly, the data were subjected to noise reduction, peak detection and selection, and then alignment following the recommendations of the xcms online tool (https://xcmsonline.scripps.edu). For the LC-MS scans in positive ionisation mode, the Agilent D files were centroided and converted into mzXML format in Agilent MassHunter Workstation software, and the peak width was set to 10–60 s to account for the wider peak widths associated with the HPLC, and the profStep function was set to 0.5 as per the xcms online tool recommendations. The phenolic dataset, consisting of the *m*/*z* values, retention times, and intensities of the putative compounds, was then analysed using the online metabolomics platform MetaboAnalyst (www.metaboanalyst.ca/). The data were normalised and log-transformed. Principal component analysis (PCA) was used to visualise the grouping of the regions based on the identified phenolic compounds. The samples included in the PCA were those sampled in the negative ionisation mode (see Table S2). Differences in anthocyanins between the regions were not assessed using PCA due to a lack of sufficient sample replicates, which would not meet statistical requirements. ANOVA followed by Fisher’s post-hoc tests were used to determine the phenolic compounds that different significantly between the regions at *p* < 0.001.

## Figures and Tables

**Table 1:** Tentative phenolic compound identifications based on the MS/MS spectra in both the negative and positive ionisation modes. Numbers **1** to **32** (negative; upper section of the table) and **1** to **9** (positive; lower section of the table) correspond to the peaks annotated in Figure 3. RT = retention time.

No.	Tentative Identification in Negative Ionisation Mode	*m*/*z* [M − H]^−^	RT	MS
**1**	Quinic acid	191.0555	1.7	173, 161, 133
**2**	Naringenin or arbutin isomer	271.0812	1.8	161, 151, 125, 119
**3**	Quinic acid derivative	317.0865	1.8	191, 179, 133
**4**	Dimer of quinic acid/caffeic acid glycoside	683.2248	1.9	341, 191
**5**	Quinic acid derivative	317.0873	5.7	191, 135 271, 255
**6**	Arbutin	271.0817	5.7	161, 109
**7**	Gallic acid	169.0137	6.3	125
**8**	3/4/5-*O*-galloylquinic acid	343.0661	7.6	301, 191, 173, 169, 125
**9**	Quinic acid derivative	405.1028	9.2	191, 173
**10**	3,4/3,5-Di*-O*-galloylquinic acid	495.0777	11.4	343, 325, 191, 169, 125
**11**	Methyl gallate	183.0297	11.7	124
**12**	Quinic acid derivative	423.0925	11.9	257, 191
**13**	1,2,3/1,4,6-Tri-*O*-galloyl-glucose	635.0884	13.4	465, 271, 169, 125
**14**	3,4,5-Tri-*O*-galloylquinic acid	647.0884	14.0	495, 343, 191, 169, 125
**15**	Ellagic acid derivative	431.1916	14.3	301, 169, 153, 125
**16**	Gallocatechin/epigallocatechin gallate	457.077	14.4	305, 169, 165, 125
**17**	Galloylquinic acid derivative	784.5787	14.8	647, 481, 301, 191, 169, 125
**18**	Quinic acid derivative	860.3122	15.4	709, 477, 301, 191
**19**	Penta-galloylquinate ester	937.0837	16.3	799, 468, 301 169, 125
**20**	Ellagic acid derivative	468.0443	16.6	301, 169, 125
**21**	Quercetin-3-O-hexose-gallate	615.1082	17.3	463, 300, 169, 151, 125
**22**	Ellagic acid derivative	441.0854	17.7	301
**23**	Ellagic acid	300.9985	18.0	229, 169, 125
**24**	Quercetin glucoside	463.0876	18.3	301
**25**	Miquelianin (quercetin-3-*O*-glucuronide)	477.0671	18.4	301, 273, 255, 179, 151
**26**	Quercetin derivative	469.0521	18.6	301
**27**	Galloyl quercetin-3-*O*-rhamnoside	599.1032	18.7	447, 301, 171, 169, 125
**28**	Digalloylglucose	483.1863	19.8	315, 301, 169, 125
**29**	Unknown phenolic	711.3957	21.0	647, 481, 301, 191, 169, 125
**30**	Quercitrin (quercetin 3-rhamnoside)	447.0923	24.0	301, 300, 271, 255, 179
**31**	Methylellagic acid pentose	447.0924	24.1	315
**32**	Isorhamnetin(3-*O*-methylquercetin)	315.0504	24.2	300, 271, 255, 243, 151
**No.**	**Tentative Identification in Positive Ionisation Mode**	***m*/*z*** **[M + H]^+^**	**RT**	**MS**
**1**	Delphinidin-3-*O*-glucoside (myrtillin)	465.1030	12.8	303, 257, 229
**2**	Delphinidin-3-(6-*O*-coumaroyl)glucoside/Delphinidin rutinoside/Quercetin 3-*O*-rutinoside (rutin)	611.1616	12.8	465, 303, 153
**3**	Quercetin-7-*O*-glucuronide (not an anthocyanin)	479.0826	13.8	303
**4**	Cyanidin-3-*O*-rutinoside	595.1667	14.8	449, 287
**5**	Delphinidin-3-*O*-arabinoside	435.0910	15.0	303
**6**	Cyanidin-3-*O*-arabinoside	419.1040	17.3	287
**7**	Peonidin-3-*O*-glucoside/galactoside	463.1242	21.0	301, 153
**8**	Petunidin-3-*O*-arabinoside	449.1079	22.4	317
**9**	Delphinidin-3-acetylglucoside	507.3283	28.8	463

## Supplementary Materials

The following are available online at https://www.mdpi.com/1420-3049/24/7/1240/s1, Figure S1: Box plots showing the abundances of the compounds (before and after transformation) deemed to differ significantly between the sampling sites at *p* < 0.001, Table S1: ANOVA and Fisher’s post-hoc test results, Table S2: Metabolomic sampling information.
