# Supplementary material for: Metabolomic Profiling of the Desiccation-Tolerant Medicinal Shrub Myrothamnus flabellifolia Indicates Phenolic Variability Across Its Natural Habitat: Implications for Tea and Cosmetics Production
Source: Molecules. 2019 Mar 29;24(7):1240. doi: 10.3390/molecules24071240 (PMC6479747; doi:10.3390/molecules24071240)
Supplement: Supplementary file 1 [file molecules-24-01240-s001.zip › molecules-450889-proof-supple-layout/molecules-450889-proof-suppl-layout-figure.pdf]

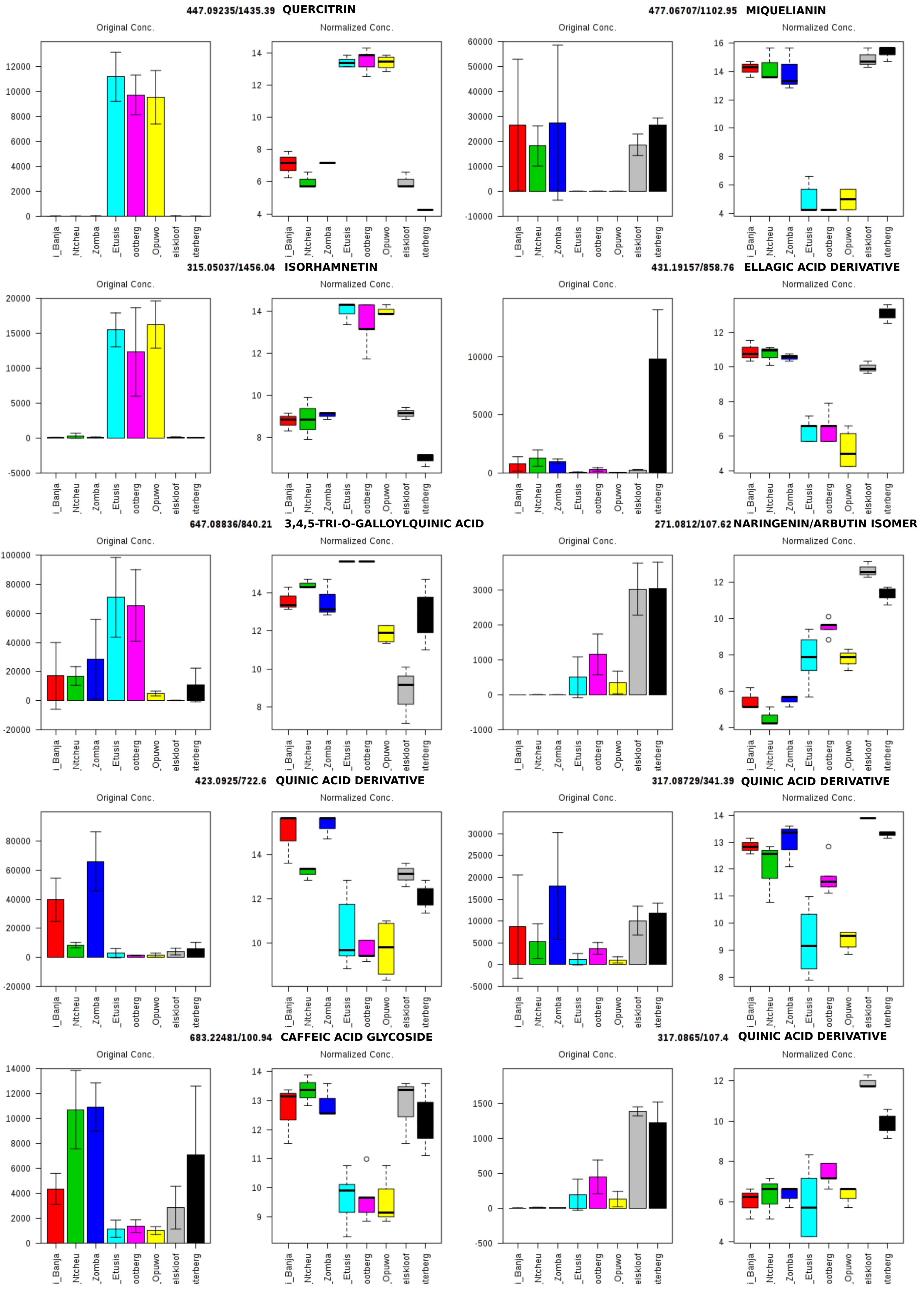
447.09235/1435.39 QUERCITRIN
477.06707/1102.95 MIQUELIANIN
315.05037/1456.04 ISORHAMNETIN
431.19157/858.76 ELLAGIC ACID DERIVATIVE
647.08836/840.21 3,4,5-TRI-O-GALLOYLQUINIC ACID
271.0812/107.62 NARINGENIN/ARBUTIN ISOMER
423.0925/722.6 QUINIC ACID DERIVATIVE
317.08729/341.39 QUINIC ACID DERIVATIVE
683.22481/100.94 CAFFEIC ACID GLYCOSIDE
317.0865/107.4 QUINIC ACID DERIVATIVE
Original Conc.
Normalized Conc.
i_Banja
_Ntcheu
_Zomba
_Etusis
ootberg
_Opuwo
elskloof
iterberg

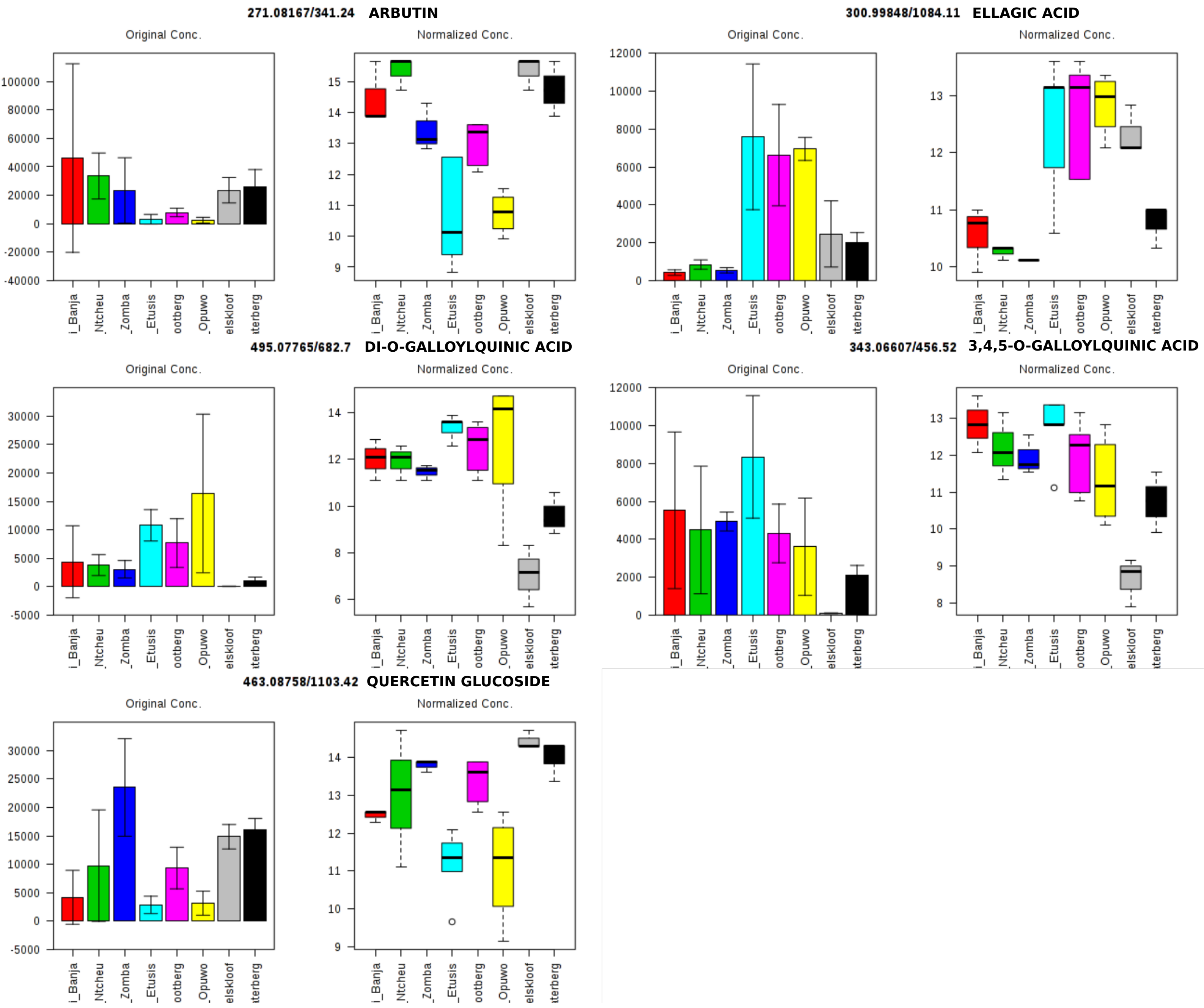


Figure S1: Box plots showing the abundances of the compounds (before and after transformation) deemed to differ significantly between the sampling sites at $p < 0.001$
