# Supplementary material for: Metabolomic Profiling of the Desiccation-Tolerant Medicinal Shrub Myrothamnus flabellifolia Indicates Phenolic Variability Across Its Natural Habitat: Implications for Tea and Cosmetics Production
Source: Molecules. 2019 Mar 29;24(7):1240. doi: 10.3390/molecules24071240 (PMC6479747; doi:10.3390/molecules24071240)
Supplement: Supplementary file 1 [file molecules-24-01240-s001.zip › molecules-450889-proof-supple-layout/molecules-450889-proof-suppl-layout-tables.pdf]

Table S1. ANOVA and Fisher's post-hoc test results.

	f.value	p.value	LOG10(p)	FDR	Fisher's LSD
447.09235/1435.39	225.44	2.6716e-18	17.573	8.2821e-17	Namibia_Etusis - Malawi_Banja; Namibia_Grootberg - Malawi_Banja; Namibia_Opuwo - Malawi_Banja; Malawi_Banja - SouthAfrica_Waterberg; Namibia_Etusis - Malawi_Ntcheu; Namibia_Grootberg - Malawi_Ntcheu; Namibia_Opuwo - Malawi_Ntcheu; Malawi_Ntcheu - SouthAfrica_Waterberg; Namibia_Etusis - Malawi_Zomba; Namibia_Grootberg - Malawi_Zomba; Namibia_Opuwo - Malawi_Zomba; Malawi_Zomba - SouthAfrica_Waterberg; Namibia_Etusis - SouthAfrica_Buffelskloof; Namibia_Etusis - SouthAfrica_Waterberg; Namibia_Grootberg - SouthAfrica_Buffelskloof; Namibia_Grootberg - SouthAfrica_Waterberg; Namibia_Opuwo - SouthAfrica_Buffelskloof; Namibia_Opuwo - SouthAfrica_Waterberg; SouthAfrica_Buffelskloof - SouthAfrica_Waterberg
477.06707/1102.95	129.57	7.9148e-16	15.102	1.2268e-14	Malawi_Banja - Namibia_Etusis; Malawi_Banja - Namibia_Grootberg; Malawi_Banja - Namibia_Opuwo; Malawi_Ntcheu - Namibia_Etusis; Malawi_Ntcheu - Namibia_Grootberg; Malawi_Ntcheu - Namibia_Opuwo; Malawi_Zomba - Namibia_Etusis; Malawi_Zomba - Namibia_Grootberg; Malawi_Zomba - Namibia_Opuwo; SouthAfrica_Buffelskloof - Namibia_Etusis; SouthAfrica_Waterberg - Namibia_Etusis; SouthAfrica_Buffelskloof - Namibia_Grootberg; SouthAfrica_Waterberg - Namibia_Grootberg; SouthAfrica_Buffelskloof - Namibia_Opuwo; SouthAfrica_Waterberg - Namibia_Opuwo
315.05037/1456.04	76.656	1.6077e-13	12.794	1.6613e-12	Namibia_Etusis - Malawi_Banja; Namibia_Grootberg - Malawi_Banja; Namibia_Opuwo - Malawi_Banja; Namibia_Etusis - Malawi_Ntcheu; Namibia_Grootberg - Malawi_Ntcheu; Namibia_Opuwo - Malawi_Ntcheu; Namibia_Etusis - Malawi_Zomba; Namibia_Grootberg - Malawi_Zomba; Namibia_Opuwo - Malawi_Zomba; Malawi_Zomba - SouthAfrica_Waterberg; Namibia_Etusis - SouthAfrica_Buffelskloof; Namibia_Etusis - SouthAfrica_Waterberg; Namibia_Grootberg - SouthAfrica_Buffelskloof; Namibia_Grootberg - SouthAfrica_Waterberg; Namibia_Opuwo - SouthAfrica_Buffelskloof; Namibia_Opuwo - SouthAfrica_Waterberg; SouthAfrica_Buffelskloof - SouthAfrica_Waterberg
431.19157/858.76	53.979	5.2301e-12	11.281	4.0533e-11	Malawi_Banja - Namibia_Etusis; Malawi_Banja - Namibia_Grootberg; Malawi_Banja - Namibia_Opuwo; Malawi_Ntcheu - Namibia_Etusis; Malawi_Ntcheu - Namibia_Grootberg; Malawi_Ntcheu - Namibia_Opuwo; SouthAfrica_Waterberg - Malawi_Ntcheu; Malawi_Zomba - Namibia_Etusis; Malawi_Zomba - Namibia_Grootberg; Malawi_Zomba - Namibia_Opuwo; SouthAfrica_Waterberg - Malawi_Zomba; SouthAfrica_Buffelskloof - Namibia_Etusis; SouthAfrica_Waterberg - Namibia_Etusis; SouthAfrica_Buffelskloof - Namibia_Grootberg; SouthAfrica_Waterberg - Namibia_Grootberg; SouthAfrica_Buffelskloof - Namibia_Opuwo; SouthAfrica_Waterberg - Namibia_Opuwo; SouthAfrica_Waterberg - SouthAfrica_Buffelskloof
271.0812/107.62	43.954	3.8833e-11	10.411	2.4076e-10	Namibia_Etusis - Malawi_Banja; Namibia_Grootberg - Malawi_Banja; Namibia_Opuwo - Malawi_Banja; SouthAfrica_Buffelskloof - Malawi_Banja; SouthAfrica_Waterberg - Malawi_Banja; Namibia_Etusis - Malawi_Ntcheu; Namibia_Grootberg - Malawi_Ntcheu; Namibia_Opuwo - Malawi_Ntcheu; SouthAfrica_Buffelskloof - Malawi_Ntcheu; SouthAfrica_Waterberg - Malawi_Ntcheu; Namibia_Etusis - Malawi_Zomba; Namibia_Grootberg - Malawi_Zomba; Namibia_Opuwo - Malawi_Zomba; SouthAfrica_Buffelskloof - Malawi_Zomba; SouthAfrica_Waterberg - Malawi_Zomba; SouthAfrica_Buffelskloof - Namibia_Etusis; SouthAfrica_Waterberg - Namibia_Etusis; SouthAfrica_Buffelskloof - Namibia_Grootberg; SouthAfrica_Buffelskloof - Namibia_Opuwo; SouthAfrica_Waterberg - Namibia_Opuwo
647.08836/840.21	25.121	7.601e-09	8.1191	3.9272e-08	Malawi_Banja - SouthAfrica_Buffelskloof; Malawi_Ntcheu - Namibia_Opuwo; Malawi_Ntcheu - SouthAfrica_Buffelskloof; Malawi_Zomba - SouthAfrica_Buffelskloof; Namibia_Etusis - Namibia_Opuwo;

					Namibia_Etusis - SouthAfrica_Buffelskloof; Namibia_Etusis - SouthAfrica_Waterberg; Namibia_Grootberg - Namibia_Opuwo; Namibia_Grootberg - SouthAfrica_Buffelskloof; Namibia_Grootberg - SouthAfrica_Waterberg; Namibia_Opuwo - SouthAfrica_Buffelskloof; SouthAfrica_Waterberg - SouthAfrica_Buffelskloof
317.08729/341.39	18.35	1.2571e-07	6.9006	5.5671e-07	Malawi_Banja - Namibia_Etusis; Malawi_Banja - Namibia_Opuwo; Malawi_Ntcheu - Namibia_Etusis; Malawi_Ntcheu - Namibia_Opuwo; Malawi_Zomba - Namibia_Etusis; Malawi_Zomba - Namibia_Opuwo; Namibia_Grootberg - Namibia_Etusis; SouthAfrica_Buffelskloof - Namibia_Etusis; SouthAfrica_Waterberg - Namibia_Etusis; Namibia_Grootberg - Namibia_Opuwo; SouthAfrica_Buffelskloof - Namibia_Opuwo; SouthAfrica_Waterberg - Namibia_Opuwo
423.0925/722.6	16.518	3.1142e-07	6.5067	1.1759e-06	Malawi_Banja - Namibia_Etusis; Malawi_Banja - Namibia_Grootberg; Malawi_Banja - Namibia_Opuwo; Malawi_Ntcheu - Namibia_Grootberg; Malawi_Ntcheu - Namibia_Opuwo; Malawi_Zomba - Namibia_Etusis; Malawi_Zomba - Namibia_Grootberg; Malawi_Zomba - Namibia_Opuwo; SouthAfrica_Buffelskloof - Namibia_Grootberg; SouthAfrica_Buffelskloof - Namibia_Opuwo
317.0865/107.4	16.341	3.4139e-07	6.4667	1.1759e-06	SouthAfrica_Buffelskloof - Malawi_Banja; SouthAfrica_Waterberg - Malawi_Banja; SouthAfrica_Buffelskloof - Malawi_Ntcheu; SouthAfrica_Waterberg - Malawi_Ntcheu; SouthAfrica_Buffelskloof - Malawi_Zomba; SouthAfrica_Waterberg - Malawi_Zomba; SouthAfrica_Buffelskloof - Namibia_Etusis; SouthAfrica_Waterberg - Namibia_Etusis; SouthAfrica_Buffelskloof - Namibia_Grootberg; SouthAfrica_Buffelskloof - Namibia_Opuwo; SouthAfrica_Waterberg - Namibia_Opuwo
683.22481/100.94	13.189	2.0371e-06	5.691	6.3151e-06	Malawi_Banja - Namibia_Etusis; Malawi_Banja - Namibia_Grootberg; Malawi_Banja - Namibia_Opuwo; Malawi_Ntcheu - Namibia_Etusis; Malawi_Ntcheu - Namibia_Grootberg; Malawi_Ntcheu - Namibia_Opuwo; Malawi_Zomba - Namibia_Etusis; Malawi_Zomba - Namibia_Grootberg; Malawi_Zomba - Namibia_Opuwo; SouthAfrica_Buffelskloof - Namibia_Etusis; SouthAfrica_Waterberg - Namibia_Etusis; SouthAfrica_Buffelskloof - Namibia_Grootberg; SouthAfrica_Waterberg - Namibia_Grootberg; SouthAfrica_Buffelskloof - Namibia_Opuwo; SouthAfrica_Waterberg - Namibia_Opuwo
271.08167/341.24	12.964	2.3435e-06	5.6301	6.6045e-06	Malawi_Banja - Namibia_Etusis; Malawi_Banja - Namibia_Opuwo; Malawi_Ntcheu - Namibia_Etusis; Malawi_Ntcheu - Namibia_Opuwo; SouthAfrica_Buffelskloof - Namibia_Etusis; SouthAfrica_Waterberg - Namibia_Etusis; SouthAfrica_Buffelskloof - Namibia_Opuwo; SouthAfrica_Waterberg - Namibia_Opuwo
300.99848/1084.11	8.0192	9.0106e-05	4.0452	0.00023277	Namibia_Opuwo - Malawi_Banja; Namibia_Etusis - Malawi_Ntcheu; Namibia_Grootberg - Malawi_Ntcheu; Namibia_Opuwo - Malawi_Ntcheu; Namibia_Etusis - Malawi_Zomba; Namibia_Grootberg - Malawi_Zomba; Namibia_Opuwo - Malawi_Zomba
495.07765/682.7	7.1827	0.00019277	3.715	0.00045968	Malawi_Banja - SouthAfrica_Buffelskloof; Malawi_Ntcheu - SouthAfrica_Buffelskloof; Malawi_Zomba - SouthAfrica_Buffelskloof; Namibia_Etusis - SouthAfrica_Buffelskloof; Namibia_Grootberg - SouthAfrica_Buffelskloof; Namibia_Opuwo - SouthAfrica_Buffelskloof
343.06607/456.52	7.0742	0.0002136	3.6704	0.00047297	Malawi_Banja - SouthAfrica_Buffelskloof; Malawi_Ntcheu - SouthAfrica_Buffelskloof; Malawi_Zomba - SouthAfrica_Buffelskloof; Namibia_Etusis - SouthAfrica_Buffelskloof; Namibia_Grootberg - SouthAfrica_Buffelskloof; Namibia_Opuwo - SouthAfrica_Buffelskloof
463.08758/1103.42	6.7628	0.0002883	3.5402	0.00059582	SouthAfrica_Buffelskloof - Namibia_Etusis; SouthAfrica_Waterberg - Namibia_Etusis; SouthAfrica_Buffelskloof - Namibia_Opuwo; SouthAfrica_Waterberg - Namibia_Opuwo

Table S2. Metabolomic sampling table.

**Metabolomic sampling table**						
**Country**	**Region**	**Accession name**	**Year collected**	**Month collected**	**Ionisation mode**	**Detection method**
Malawi	Zomba plateau	ZOM37	2015	April	Negative	LC-MS/MS
Malawi	Zomba plateau	ZOM38	2015	April	Positive	LC-MS
Malawi	Zomba plateau	ZOM40	2015	April	Negative	LC-MS/MS
Malawi	Zomba plateau	ZOM41	2015	April	Negative	LC-MS/MS
Malawi	Ntcheu	NT43	2015	April	Negative	LC-MS/MS
Malawi	Ntcheu	NT44	2015	April	Positive	LC-MS
Malawi	Ntcheu	NT45	2015	April	Negative	LC-MS/MS
Malawi	Ntcheu	NT49	2015	April	Negative	LC-MS/MS
Malawi	Banja Hill	BAN51	2015	April	Positive	LC-MS/MS
Malawi	Banja Hill	BAN52	2015	April	Negative	LC-MS/MS
Malawi	Banja Hill	BAN54	2015	April	Negative	LC-MS/MS
Malawi	Banja Hill	BAN55	2015	April	Negative	LC-MS/MS
Namibia	Opuwo	OP1	2014	October	Negative	LC-MS/MS
Namibia	Opuwo	OP2	2014	October	Positive	LC-MS/MS
Namibia	Opuwo	OP3	2014	October	Negative	LC-MS/MS
Namibia	Opuwo	OP4	2014	October	Negative	LC-MS/MS
Namibia	Opuwo	OP5	2014	October	Negative	LC-MS/MS
Namibia	Grootberg	GB10	2014	October	Positive	LC-MS
Namibia	Grootberg	GB11	2014	October	Negative	LC-MS/MS
Namibia	Grootberg	GB13	2014	October	Negative	LC-MS/MS
Namibia	Grootberg	GB19	2014	October	Positive	LC-MS/MS
Namibia	Grootberg	GB22	2015	August	Negative	LC-MS/MS
Namibia	Grootberg	GB27	2015	August	Negative	LC-MS/MS
Namibia	Grootberg	GB28	2015	August	Negative	LC-MS/MS
Namibia	Etusis	ET17	2014	October	Negative	LC-MS/MS
Namibia	Etusis	ET20	2014	October	Negative	LC-MS/MS
Namibia	Etusis	ET23	2014	October	Positive	LC-MS
Namibia	Etusis	ET25	2014	October	Negative	LC-MS/MS
Namibia	Etusis	ET29	2015	August	Positive	LC-MS/MS
Namibia	Etusis	ET30	2015	August	Negative	LC-MS/MS
Namibia	Etusis	ET34	2015	August	Negative	LC-MS/MS
South Africa	Waterberg	JILL57	2015	July	Positive	LC-MS
South Africa	Waterberg	JILL58	2015	July	Negative	LC-MS/MS
South Africa	Waterberg	JILL59	2015	July	Negative	LC-MS/MS
South Africa	Waterberg	JILL61	2015	July	Negative	LC-MS/MS
South Africa	Buffelskloof	BK1	2015	September	Negative	LC-MS/MS
South Africa	Buffelskloof	BK5	2016	July	Negative	LC-MS/MS
South Africa	Buffelskloof	BK6	2016	July	Negative	LC-MS/MS
